# Dual-Pulse Repeated Frequency Waveform Design of Time-Division Integrated Sensing and Communication Based on a 5G New Radio Communication System

**DOI:** 10.3390/s23239463

**Published:** 2023-11-28

**Authors:** Ping Chu, Zhaocheng Yang, Jian Zheng

**Affiliations:** College of Electronics and Information Engineering, Shenzhen University, Shenzhen 518060, China; chuping@szu.edu.cn (P.C.); 2110436094@email.szu.edu.cn (J.Z.)

**Keywords:** integrated sensing and communication, 5G NR, dual-PRF, waveform design, range and velocity estimation

## Abstract

With the development of 5G communication systems, it is a hot topic to embed integrated sensing and communication (ISAC) based on the existing 5G base station by sharing the hardware and the same frequency spectrum. In this paper, we propose a dual pulse repeated frequency (dual-PRF) waveform design of time-division ISAC (TD-ISAC) based on a 5G new radio (NR) communication system using downlink communication slots. We choose time-division mode to design waveform to avoid the interference between sensing and communication. Embedding sensing functions in a 5G NR system, we design a dual-PRF sensing slot to satisfy the constraints of common channel and uplink communication. Considering two uplink modes, namely flexible and fixed, we design two dual-PRF waveforms and illustrate the sensing theory performance of the designed waveform by the ambiguity function. Then, we exploit the designed waveform to the vehicle parameter estimation. To verify that the designed waveform has good adaptability to different signal processing methods, we realize the parameter estimation by two types of methods: the discrete Fourier transformation-based method and the compressed sensing-based method. At last, we verify the effectiveness of the designed waveform system by simulation experiments and real traffic scenarios.

## 1. Introduction

Over the past several years, integrated sensing and communications (ISAC) has attracted widespread attention. ISAC can reduce hardware costs and greatly improve frequency spectrum efficiency by reusing hardware and the same frequency spectrum [1,2,3]. As popularly seen in the literature, ISAC has broad applications, such as autonomous driving vehicles [4], vehicle-to-vehicle [5] systems, indoor locations [6], wireless radar sensor networks [7], and drone surveillance networks [8].

The main research work on ISAC is how to design the waveform to obtain good performance of both sensing and communication. Generally, the waveform design of ISAC can be mainly divided into three categories: sensing-centered [9,10], communication-centered [11,12], and joint design [13,14]. The sensing-centered methods add communication functions by reusing the existed sensing system. It provides a signal processing perspective of mm-wave ISAC systems with an emphasis on waveform design in [15]. The communication-centered methods are contrasted to sensing-centered methods in that they add sensing functions by reusing the existed communication system. Sensing mutual information (MI) is applied as a performance metric to optimize the waveform by adding sensing paddings in the idle communication layers without degrading communication performance [16]. Based on the classical phase-coded waveform used in wireless communication, ref. [17] studied its requirements for high dynamic range radar measurement, and discussed a variety of possible radar processing algorithms. Based on the millimeter-wave wireless local area network standard, ref. [18] designed the joint waveform of automotive radar and potential millimeter-wave vehicle communication systems. The performance of the uplink integrated sensing and communication system in which communication users and radar targets share the same frequency band is analyzed in [19], and new expressions are derived to characterize the outage probability, ergodic communication rate, and sensing rate. In order to break the limitation of the cyclic prefix (CP) on the sensing range, the echo signal is evenly divided into sub-blocks in [20]. For each sub-block, the virtual cyclic prefix method is used to remove the communication data symbol, and two types of range-Doppler maps are generated for sensing.

The sensing-centered methods face the problem of low communication rate, while the communication-centered methods bring problems such as peak-to-average-power patio (PAPR) and random autocorrelation due to the randomness of communication symbols. Hence, it is necessary to jointly optimize the waveform design of ISAC to balance the performance of the two functions. A sensing integrated discrete Fourier transform spread orthogonal frequency division multiplexing (SI-DFT-s-OFDM) system is proposed for THz ISAC, which can provide a lower PAPR power ratio than OFDM and is adaptive to a flexible delay spread of the THz channel [21]. In [22], a novel waveform design method for Full-Duplex integration is proposed. The communication signal is transmitted by using the idle time of the traditional pulse radar, and can improve the detection probability of the sensing target and the communication rate of the system. It is usually required to have excellent self-ambiguity function and cross-ambiguity function to improve the performance of measurement accuracy and the suppression of clutter interference. Ref. [23] designed a synthesis signal with a strict low ambiguity function to improve sensing performance.

With the rapid development of 5G technology, 5G communication systems are being widely commercially deployed. Especially, orthogonal frequency division multiplexing (OFDM) waveforms used in 5G communication systems provide high communication data rates and the ability to efficiently deal with frequency selective fading. Ref. [24] verifies the performance benefit and feasibility to enable ISAC in 5G for the sensing mode based on the base station. Ref. [25] explores different properties of signals available for radar sensing and aims to combine the ISAC technology with the cellular network by optimizing the multi-dimensional resource scheduling. The joint state estimation problem for unmanned aerial vehicle (UAV)-aided 6G communications is considered in [26], and the channel characteristics of the spatial channel are analyzed. Ref. [27] optimizes the ISAC waveform by filling the empty subcarriers of a 5G base station working in downlink mode and optimizing the transmission power of some of the communication subcarriers. However, the communication waveform is random in nature. And the number of empty subcarriers in the communication depends on the number of users served by the networks. The data transmitted by the users has burst and discontinuity. Ref. [28] proposes a novel ISAC scheme which constructs three adjacent base stations as a cooperative sensing system. But this scheme would reduce the duration in downlink mode by one-third, which would significantly reduce the communication data rate. Therefore, how to reuse the hardware of existing widely deployed 5G base stations to achieve ISAC while minimizing the impact on the existing communication data rate is an important problem.

It can be seen from the above literature that the waveform design of ISAC is usually uniform. In fact, it cannot realize uniform sensing in a 5G NR communication system because the waveform design of ISAC in a 5G NR communication system needs to satisfy the constraints of the communication protocol which contains uplink and common channel. However, a nonuniform sensing waveform leads to nonuniform sampling. And the uniform signal processing method cannot work. Its ambiguity function has some big side-lobes which will affect its estimation performance. And the FFT method can work no longer under the nonuniform distribution. On the other hand, the millimeter wave band in 5G greatly reduces the time of each communication symbol [29,30]. Hence, utilizing a small number of symbols in one frame for sensing can greatly alleviate the problem of low efficiency.

In this paper, we design a TD-ISAC sensing waveform based on a 5G communication base station with its millimeter wave band, which realizes sensing by reusing the hardware and OFDM waveform of an existing 5G NR communication base station. That is, we realize ISAC by reusing hardware resources. And the designed sensing waform reuses the millimeter wave of the 5G NR communication with OFDM waveform. That is, we realize ISAC by reusing communication spectrum and waveform. Furthermore, to avoid the interference between sensing and communication, we choose the time-division mode to design the waveform. And we allocate one OFDM symbol for sensing in one time slot at most to deduce the impact on communication efficiency. Then, we design two dual-PRF waveforms under the constraints of communication protocol, and the designed waveform is nonuniform adaptive to the real communication scenarios. To verify that the designed waveform has good adaptability to different signal processing methods, we realize the parameter estimation by two types of methods: the discrete Fourier transformation-based method and the compressed sensing-based method. Finally, we verify the effectiveness of the designed waveform in vehicle sensing applications by simulation and real traffic experiments.

## 2. Signal Model and Problem Formulation

In this section, we introduce the ISAC signal model with the OFDM waveform in a 5G communication system, and we detail the waveform design motivation and constraints.

### 2.1. ISAC Signal Model with OFDM Waveform

OFDM waveform is generally used to transmit signals in a 5G communication system. Considering the millimeter waveband in 5G, the OFDM communication symbols can be shared for sensing. In other words, an ISAC system can be constructed based on the 5G communication system. Analytically, the transmitted OFDM signal can be expressed as

(1) $$s\left(t\right)=\sum_{m=0}^{N_{s}-1}\sum_{n=0}^{N_{c}-1}a_{n,m}e^{j2\pi f_{c}t}e^{j2\pi n\Delta f(t-mT_{s})}\mathrm{rect}\left(\frac{t-mT_{s}}{T_{s}}\right),$$

where $N_{s}$ denotes the number of OFDM symbols composing the communication frame, $N_{c}$ denotes the total amount of subcarries, $a_{n,m}$ denotes the nth complex modulation information of the mth OFDM symbol, nΔf is the nth individual subcarrier frequency, $f_{c}$ denotes the carrier frequency, $T_{s}=T_{cp}+T$ denotes the total OFDM symbol duration composed of an elementary symbol duration T=1/Δf and a CP duration $T_{cp}$, and $\mathrm{rect}(t/T_{s})$ represents a rectangular window of duration $T_{s}$.

Considering an object in the range r with velocity v, the received baseband signal can be described as

(2) $$\begin{array}{c} \begin{array}{cc} y(t) & =y_{t}\left(t\right)+y_{c}\left(t\right)+y_{n}\left(t\right)\\ & =\sigma \sum_{m=0}^{N_{s}-1}e^{j2\pi f_{d}mT_{s}}\sum_{n=0}^{N_{c}-1}a_{n,m}e^{j2\pi n\Delta f(t-mT_{s})}e^{-j2\pi n\Delta f\tau }\mathrm{rect}\left(\frac{t-mT_{s}-\tau }{T_{s}}\right)+y_{c}\left(t\right)+y_{n}\left(t\right), \end{array} \end{array}$$

where $y_{t}\left(t\right)$ denotes the object reflection signal, $y_{c}\left(t\right)$ denotes the clutter due to the reflection of ground, green belts, and wind, etc., $y_{n}\left(t\right)$ denotes the receiver thermal noise, σ denotes attenuation and phase shift occurring due to the propagation and scattering process, and $f_{d}$ and τ denote the Doppler shift and the round-trip propagation time of the object, respectively. They can be described by

(3) $$f_{d}=2vf_{c}/c,$$

with *c* being the speed of light, and

(4) τ=2r/c.

In order to describe the signal processing conveniently, we only rewrite the object reflection signal here. Then, according to (2), the mth received OFDM symbol with removing CP can be rewritten as

(5) $$y_{tm}\left(t\right)=\sigma e^{j2\pi f_{d}mT_{s}}\sum_{n=0}^{N_{c}-1}a_{n,m}e^{j2\pi n\Delta f(t-mT_{s})}e^{-j2\pi n\Delta f\tau }.$$

Obviously, we can separate subcarrier of the *m*th OFDM symbol by exploiting DFT with sampling interval $1/N_{c}\Delta f$. Specifically, the modulation information can be removed by an element-wise complex division to $a_{n,m}$. Then, the *m*th received OFDM symbols can be described as a vector

(6) $$\begin{array}{c} \begin{array}{c} y_{tm}=\sigma e^{j2\pi f_{d}mT_{s}}\left[1,e^{-j2\pi \Delta f\tau },\ldots ,e^{-j2\pi (N_{c}-1)\Delta f\tau }\right]. \end{array} \end{array}$$

We perform the same operation on each received OFDM symbol in (2), and can obtain the received matrix expressed as

(7) $$\begin{array}{c} \begin{array}{cc} Y & ={\left[y_{t0}^{T},y_{t1}^{T},\ldots ,y_{t(N_{s}-1)}^{T}\right]}^{T}+Y_{c}+Y_{n}\\ & =\sigma b_{fd}⊗b_{\tau }+Y_{c}+Y_{n}, \end{array} \end{array}$$

where $Y_{c}$ denotes the clutter matrix, $Y_{n}$ denotes the thermal noise matrix, $b_{fd}$ denotes the Doppler dimension vector, and $b_{\tau }$ denotes the range dimension vector, and they are expressed as

(8) $$\begin{array}{c} \begin{array}{c} b_{fd}={\left[1,e^{j2\pi f_{d}T_{s}},\ldots ,e^{j2\pi f_{d}\left(N_{s}-1\right)T_{s}}\right]}^{T}, \end{array} \end{array}$$

and

(9) $$\begin{array}{c} \begin{array}{c} b_{\tau }=\left[1,e^{-j2\pi \Delta f\tau },\ldots ,e^{-j2\pi (N_{c}-1)\Delta f\tau }\right]. \end{array} \end{array}$$

According to (9), we can conveniently evaluate τ by computing the $N_{c}$-points inverse discrete Fourier transform (IDFT) of (7) along each row of Y. In a similar way, the determination of $f_{d}$ can be solved by applying a $N_{s}$-points DFT along each column of Y. Furthermore, we can compute the range r and the velocity v of the reflected object conveniently according to (3) and (4).

### 2.2. Motivation and Problem Statement

**Figure 1:**
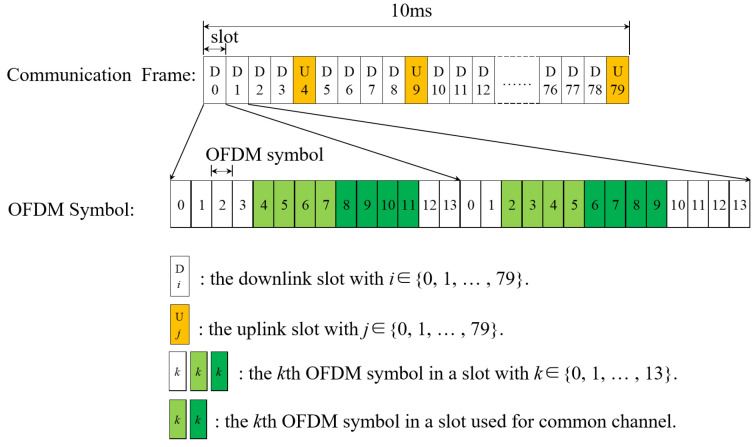
5G NR communication frame structure.

As described in the previous section, an ISAC system can be designed for vehicle sensing under 5G communication base stations. However, the distribution of sensing symbols needs to be subject to the constraints of the 5G NR communication frame structure as shown in Figure 1, which takes subcarrier interval Δf = 120 kHz, for example. Generally, there are a total of 80 slots in a frame with settable downlink slot and uplink slot, which are allocated by the communication system parameters, and each slot contains 14 OFDM symbols. Obviously, the 5G NR frame structure will lead to a nonuniform distribution of sensing symbols. The reasons are as follows: (1) the communication frame structure contains uplink slots. However, the object sensing is realized based on the downlink signal. Therefore, it cannot satisfy the timing uniform structure of (9); and (2) the communication frame structure contains a common channel and it needs to occupy partial subcarriers in some OFDM symbols. Then, it cannot satisfy the subcarrier uniform structure of (8) with the common channel [31].

Due to the above two nonuniform structures, ISAC cannot work well with the existing time division duplex 5G NR communication base station. Otherwise, the sharing model of communication symbols and sensing symbols leads to the problems of interference and high peak side-lobe ratio (PSLR) [32]. Therefore, we propose the TD-ISAC based on a 5G NR communication base station. That is, we allocate some OFDM symbols in a communication frame designed especially for sensing. Furthermore, we use fewer OFDM symbols for sensing in order to keep a high communication efficiency. In this paper, the waveforms are designed under the following assumptions.

As1: Sensing symbol cannot be allocated at uplink slot. There is one uplink slot in every five consecutive slots satisfying the max uplink demand in a 5G NR communication protocol. And the uplink slot can be set in any slot. If the uplink slot is set in any slot as flexible mode, we call it uplink-flexible mode. On the contrary, if the uplink slot is set in one fixed position, we call it uplink-fixed mode.

As2: One sensing symbol can be allocated at most in a slot under uplink-flexible mode, and it can be slightly adjusted under uplink-fixed mode.

As3: Sensing symbol cannot be allocated at common channel.

**Figure 2:**
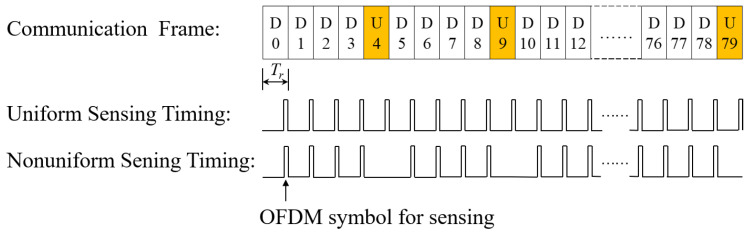
Sensing symbol timing (where the orange color represents uplink slot).

As shown in Figure 1, the common channel occupies only partial OFDM symbols in one slot. Considering the assumption As2 and As3, we allocate the 13th OFDM symbol in a downlink slot for sensing. Then, the nonuniform sensing symbol timing can be illustrated as Figure 2, where the uniform sensing timing represents the ideal situation with no assumption considering for comparison. Obviously, the sensing symbol timing in the TD-ISAC system is similar to the radar sensing pulse. And $T_{r}$ is the pulse repetition interval (PRI) of the uniform sensing timing equal to one slot time.

Then, $b_{fd}$ of the uniform sensing timing in the received matrix can be rewritten as

(10) $$\begin{array}{c} \begin{array}{c} {\mathrm{bu}}_{fd}={\left[1,\ldots ,e^{j2\pi f_{d}kT_{r}},\ldots ,e^{j2\pi f_{d}\left(N_{u}-1\right)T_{r}}\right]}^{T}, \end{array} \end{array}$$

where $N_{u}$ is the total number of uniform sensing symbols and $k=\left\{0,1,\ldots ,(N_{u}-1)\right\}$. Let $J=\left[j\left(0\right),\ldots ,j\left(i\right),\ldots ,j\left(N_{n}-1\right)\right]$ be the selected matrix, where $j\left(i\right)={\left[0,\ldots ,1,\ldots ,0\right]}^{T}$ with almost 0 except for the kth element of the uniform sensing symbol corresponding to the ith nonuniform sensing symbol. And $N_{n}$ is the total number of nonuniform sensing symbols. Then, $b_{fd}$ of the nonuniform sensing timing in the received matrix can be rewritten as

(11) $$\begin{array}{c} \begin{array}{c} {\mathrm{bn}}_{fd}={\mathrm{bu}}_{fd}J. \end{array} \end{array}$$

In this paper, we will design the nonuniform sensing timing of TD-ISAC for vehicle sensing based on the 5G base station under urban traffic scenarios in the following section.

## 3. Dual-PRF Waveform Design and Signal Processing

In this section, we first design two types of nonuniform sensing timings under uplink-flexible and uplink-fixed mode, respectively. And then we verify the theory performance by some experiments. At last, we detail the sensing signal processing for the designed waveform.

According to the nonuniform of the 5G NR communication system, we propose a dual-PRF waveform design, which realizes sensing by reusing the hardware and OFDM waveform of an existing 5G NR communication base station. First, the sensing waveform and communication waveform work under time division mode. Hence, the two cannot interfere each other; that is, the sensing waveform does not impact the quality of the communication service. Second, in the 5G NR communication system, each symbol lasts for a very short time with the millimeter wave band. Hence, utilizing a small number of symbols in one frame for sensing within the millimeter wave band has very little effect on communication efficiency. More precisely, we allocate only one OFDM symbol for sensing in one time slot at most to deduce the impact on communication efficiency.

Furthermore, according to the 5G NR communication protocol, there is some uplink slots in one frame. And one uplink slot in every five consecutive communication slots can satisfy the max uplink demand. The uplink slot can be set in any slot or fixed position among the five consecutive slots. In order to adapt the two uplink communication modes, we designed a sensing waveform under uplink-flexible mode and uplink-fixed mode, respectively. The uplink-flexible mode is called when the uplink position is flexible in every five consecutive slots. On the contrary to flexible mode, if the uplink position is fixed in one slot of every five consecutive slots, we call it uplink-fixed mode.

As we know, if we estimate vehicle parameters by applying DFT to (8) and (9), the range resolution Δr can be computed by

(12) $$\Delta r=\frac{c}{2B}=\frac{c}{2N_{c}\Delta f}$$

where B denotes the bandwidth. Then, the maximum unambiguity range $r_{u}$ can be computed by

(13) $$r_{u}=\frac{c\tau_{u}}{2}=\frac{c}{2\Delta f}.$$

In this paper, we take for example subcarrier interval Δf = 120 kHz and B = 400 MHz. Then, we can obtain the range resolution Δr = 0.375 m and the maximum unambiguity range $r_{u}$ = 1.25 km. Generally, $T_{\mathrm{frame}}$ = 10 ms, and considering $f_{c}$ = 26 GHz, we can compute the velocity resolution Δv as

(14) $$\Delta v=\frac{c}{2f_{c}}\Delta f_{d}=\frac{c}{2f_{c}}\frac{1}{T_{frame}}=2.08\;\mathrm{km}/h.$$

Furthermore, according to the above parameters, the unambiguity velocity $v_{u}$ of the uniform sensing timing can be computed as

(15) $$v_{u}=\pm \frac{c}{2f_{c}}f_{du}=\pm \frac{c}{2f_{c}}\frac{1}{2T_{r}}=\pm 83.08\;\mathrm{km}/h.$$

Obviously, the uniform sensing timing can satisfy the vehicle sensing by applying DFT conveniently under urban traffic scenarios. According to the descriptions of TD-ISAC, above all it can be seen that the nonuniform sensing timing does not change the uniform structure of (8). Hence, in one communication frame, it can also satisfy the vehicle sensing parameter of Δr, $r_{u}$ and Δv except $v_{u}$. Therefore, we mainly consider the timing design of the nonuniform sensing pulse corresponding to $f_{d}$ of the reflected object.

### 3.1. Waveform Design under Uplink-Flexible Mode

As shown in Figure 2, the sensing timing of TD-ISAC is similar to the radar sensing pulse. We design nonuniform sensing timing based on the idea of ambiguity resolution by multiple PRF in a radar system. It can be seen from Figure 1 that the common channel symbols are in the middle of some slots with some reserved OFDM symbols at the head or end of the slots. Hence, we avoid the common channel by allotting a sensing symbol at the last position of one slot.

**Figure 3:**
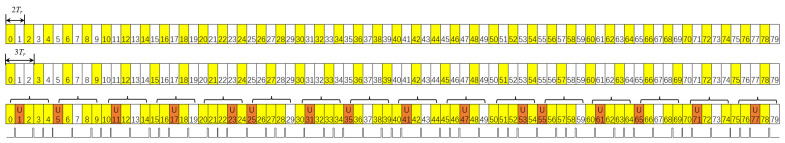
Dual-PRF sensing timing under uplink-flexible mode.

**Figure 4:**
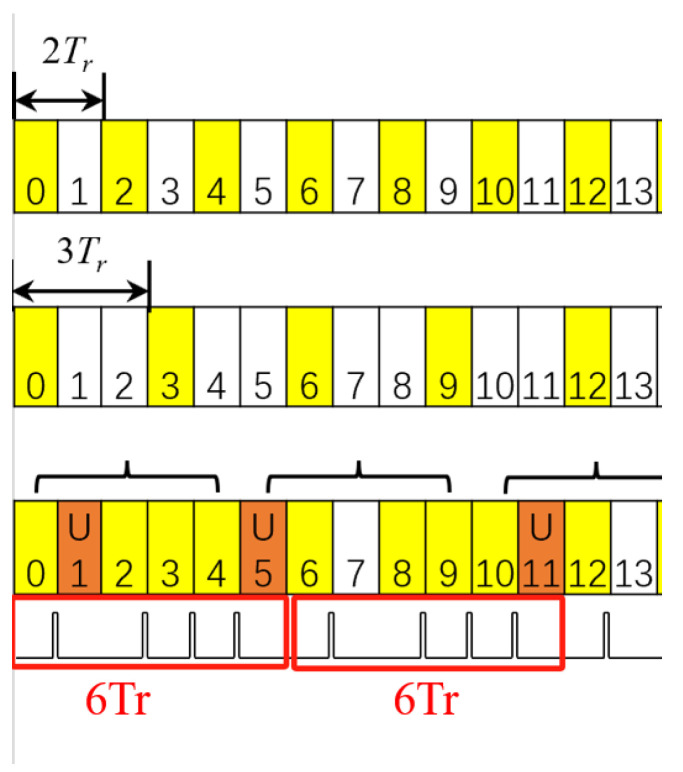
Part of designed waveform.

Here, we first consider uplink-flexible mode. According to As1, one uplink slot needs to be allocated in every five consecutive slots. We select the dual-PRF method to design the sensing waveform. Figure 3 shows the designed pulse, where the color of yellow represents the last symbol of the slot is allocated for sensing. It can be seen from the figure that there is at least one slot free for uplink in every five consecutive slots which satisfies the assumption As1. The designed dual-PRF sensing waveforms are nonuniform, and the dual-PRFs are 2$T_{r}$ and 3$T_{r}$, respectively. We know the least common multiple of 2$T_{r}$ and 3$T_{r}$ is 6$T_{r}$. Obviously, there are two idle symbols in every six symbols as shown in Figure 4; that is, the sensing idle is 1/3. The maximum uplink demand is one slot in five consecutive slots. Obviously, 1/3 > 1/5, the sensing idle of the designed waveform can satisfy the maximum link needs. And from the designed waveform in Figure 3, we can see that the designed sensing waveform satisfies the maximum link needs which is assumed in As3.

The 2$T_{r}$ and 3$T_{r}$ pulses are both uniform pulses. Similar to (10), the Doppler dimension vector of the two pulses in the received matrix can be respectively expressed as

(16) $$\begin{array}{c} \begin{array}{c} b_{2flx}=\left[1,\ldots ,e^{j2\pi f_{d}k2T_{r}},\ldots ,e^{j2\pi f_{d}\left(N_{2T}-1\right)2T_{r}}\right] \end{array} \end{array}$$

and

(17) $$\begin{array}{c} \begin{array}{c} b_{3flx}=\left[1,\ldots ,e^{j2\pi f_{d}l3T_{r}},\ldots ,e^{j2\pi f_{d}\left(N_{3T}-1\right)3T_{r}}\right], \end{array} \end{array}$$

where *k* is the *k*th pulse of the 2$T_{r}$ sensing waveform, *l* is the *l*th pulse of the 3$T_{r}$ sensing waveform, and $N_{2T}$, $N_{3T}$ are the number of 2$T_{r}$ and 3$T_{r}$ pulses. Then, the unambiguity velocities with the DFT-based method of the two pulses are

(18) $$v_{u2Tr}=\pm \frac{c}{2f_{c}}\frac{1}{4T_{r}}=\pm 41.5\;\mathrm{km}/h$$

and

(19) $$v_{u3Tr}=\pm \frac{c}{2f_{c}}\frac{1}{6T_{r}}=\pm 27.7\;\mathrm{km}/h,$$

respectively. Neither the 2$T_{r}$ nor the 3$T_{r}$ pulse can satisfy the unambiguity velocity in urban traffic. However, based on the dual-PRF ambiguity resolution method, it can obtain the unambiguity velocity [33]

(20) $$v_{uDPRF}=\pm \frac{c}{2f_{c}}\frac{1}{2\left(3T_{r}-2T_{r}\right)}=\pm 83.08\;\mathrm{km}/h$$

Obviously, it can obtain the same unambiguity velocity with uniform sensing timing.

### 3.2. Waveform Design under Uplink-Fixed Mode

**Figure 5:**
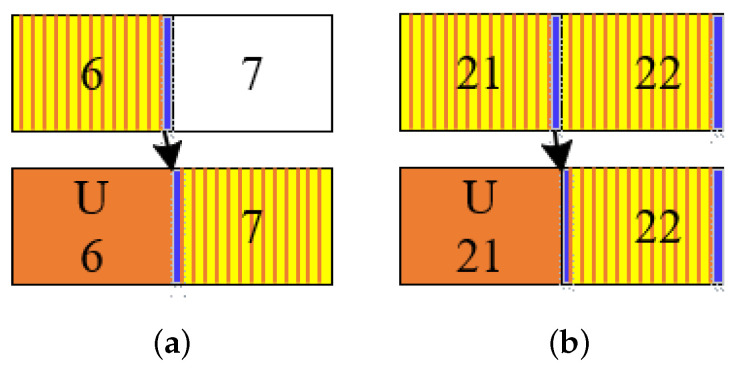
The timing modification case. (**a**) The 6th modification. (**b**) The 21th modification.

**Figure 6:**
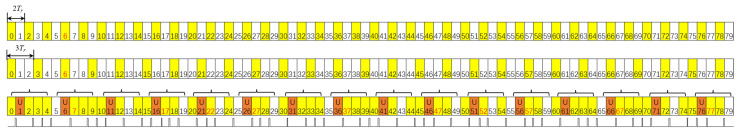
Dual-PRF sensing timing under uplink-fixed mode.

As shown in Figure 3, the uplink slot in every five consecutive slots is varying under uplink-flexible mode. In order to simplify the structure of TD-ISAC, we then modify the sensing timing based on the uplink-flexible mode with the constraints of uplink-fixed. Without loss of generality, we assume that the second slot in every five consecutive slots is fixed as uplink slot. Then, there are some slot conflicts that sensing symbols occupy the fixed uplink slot, such as 6, 16, 21, 26, 41, 46, 51, 56, 66, and 76. We move the sensing symbol of the conflict slot to the first OFDM symbol of the next slot in order to change less the structure as shown in Figure 5, where blue represents the OFDM symbol allocated for sensing, yellow represents part of the slot allocated for sensing, and orange represents the uplink slot (which is the same meaning as in the following text). Taking the sixth slot, for example, it needs to be set as uplink slot in uplink-fixed mode, and then its sensing symbol is moved to the first symbol of the seventh slot as shown in Figure 5a. Similarly, the twenty-first slot needs to be set as uplink slot in uplink-fixed mode, and then its sensing symbol is moved to the first symbol of the twenty-second slot as shown in Figure 5b. The difference between the two cases is that the seventh slot does not contain any sensing symbol in uplink-flexible mode. Hence, it has one sensing symbol in the uplink-fixed mode, while the twenty-first slot contains one sensing symbol in uplink-flexible mode, resulting in two sensing symbols in the uplink-fixed mode. Applying the same operation to all conflict slots, we can then obtain the sensing timing under uplink-fixed mode, as illustrated in Figure 6. Compared with sensing timing under uplink-flexible mode, the 2Tr and 3Tr pulses are no longer strict uniform pulses with a small time error that can be tolerated. Its theoretical performance will be analyzed in the next part. Similarly to (16) and (17), the Doppler dimension vector of the 2$T_{r}$ and 3$T_{r}$ pulses in the received matrix can be expressed as

(21) $$\begin{array}{c} \begin{array}{c} b_{2fix}=b_{2flx}⊙\left[1,1,\ldots ,e^{j2\pi f_{d}k_{m}2T_{r}T_{s}},\ldots \right], \end{array} \end{array}$$

and

(22) $$\begin{array}{c} \begin{array}{c} b_{3fix}=b_{3flx}⊙\left[1,1,\ldots ,e^{j2\pi f_{d}l_{m}3T_{r}T_{s}},\ldots \right], \end{array} \end{array}$$

where ⊙ denotes Hadamard product, $k_{m}$ denotes the number of conflict slot modifications of the 2$T_{r}$ sensing pulse, and $l_{m}$ denotes the number of the conflict slot modifications of the 3$T_{r}$ sensing pulse.

During the waveform-designed process, we avoid the common channel by allotting a sensing symbol at the last position of one slot under uplink-flexible mode. There are some symbol modifications under uplink-fixed mode, and we shift the symbol to the first symbol of the next slot. It is also allocated at the idle OFDM symbols, so the proposed waveform can satisfy the constraint of common channel which is assumed in As3. And from the designed waveform in Figure 6 where the red number represents the modification position compare with the sensing timing under uplink-flexible mode. We can see that the designed sensing waveform satisfies the maximum uplink needs that is one slot in five consecutive slots.

### 3.3. Theory Analysis of the Designed Waveform

In this section, we assess the sensing theory performance of the designed waveform by the ambiguity function with its PSLR and integrated side-lobe ratio (ISLR). And the ambiguity function can be expressed as

(23) $$\begin{array}{cc} \chi_{u}\left(\tau ,f_{d}\right)= & \int_{-\infty }^{\infty }s^{H}\left(t-\tau \right)s_{t}e^{j2\pi f_{d}t}dt\\ = & \sum_{l=0}^{L-1}\sum_{n=0}^{N_{c}-1}\sum_{n^{\prime }=0}^{N_{c}-1}(T_{s}-\left|\tau |\right)e^{j\pi \left[\left(n-n^{\prime }\right)\Delta f+f_{d}\right]\left(T_{s}+\tau \right)}\\ \; & \cdot \;e^{j2\pi n^{\prime }\Delta f\tau }\mathrm{sinc}\left\{\pi \left[\left(n-n^{\prime }\right)\Delta f+f_{d}\right](T_{s}-\left|\tau |\right)\right\}e^{j2\pi f_{d}lT_{s}}, \end{array}$$

where *L* denotes the number of OFDM symbols.

**Figure 7:**
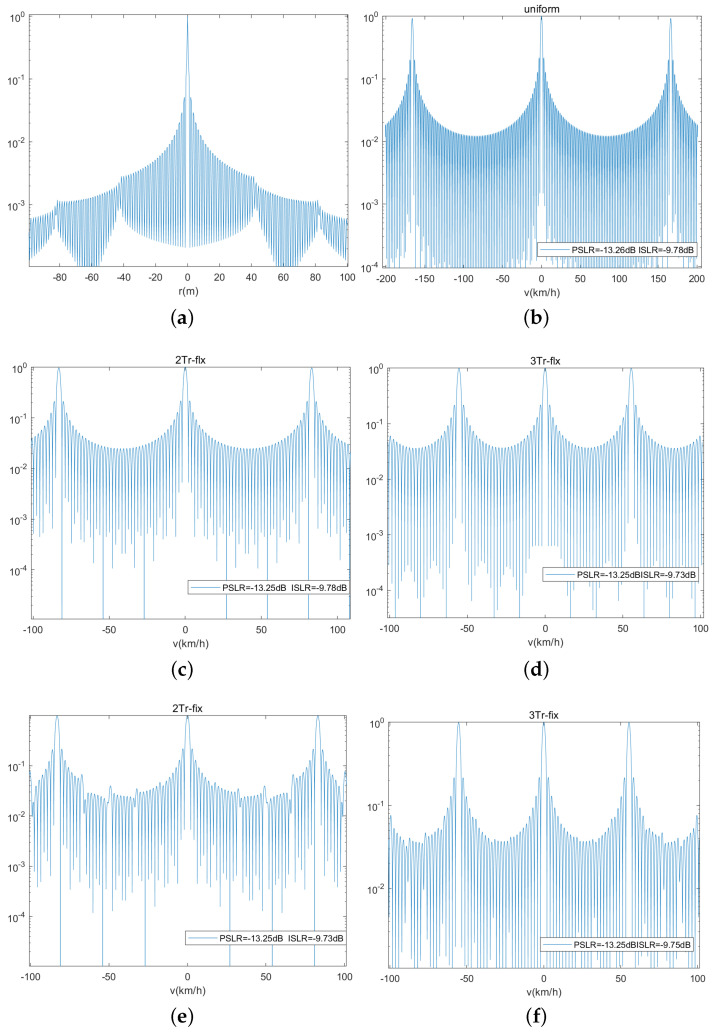
Ambiguity function of different waveforms. (**a**) Range ambiguity function. (**b**) Velocity ambiguity function of uniform pulse. (**c**) Velocity ambiguity function of 2$T_{r}$ under uplink-flexible mode. (**d**) Velocity ambiguity function of 3$T_{r}$ under uplink-flexible mode. (**e**) Velocity ambiguity function of 2$T_{r}$ under uplink-fixed mode. (**f**) Velocity ambiguity function of 3$T_{r}$ under uplink-fixed mode.

The numeral experiments are obtained under parameters involving 5G communication subcarrier interval Δf = 120 kHz, bandwidth B = 400 MHz, and carrier frequency $f_{c}$ = 26 GHz. Figure 7 shows the ambiguity function of uniform sensing, dual-PRF sensing timing under uplink-flexible mode, and uplink-fixed mode, respectively. The range r of the reflected object can be estimated by the CP duration $T_{cp}$ in the 5G NR communication with OFDM waveform. And its range can be detected at 87 m with the assumed parameters. The difference between the three waveforms is the Doppler dimension. So they have the same range ambiguity function, and Figure 7a shows that they can estimate r unambiguity within the detected range. The uniform sensing ambiguity function of v is shown in Figure 7b with unambiguity v=±83.08 km/h. Figure 7c,d shows the velocity ambiguity function of 2Tr and 3Tr under uplink-flexible mode. It can be seen that their unambiguity velocities are ±41.5 km/h and ±27.7 km/h. Similarly, Figure 7e,f are the velocity ambiguity functions of 2Tr and 3Tr under uplink-fixed mode. It can be also seen that, comparing Figure 7c,d with Figure 7e,f, the sidelobe of the velocity ambiguity function under the uplink-flexible mode is smoother than that of the uplink-fixed mode.

### 3.4. Signal Processing

In this subsection, we introduce the signal processing methods of the dual-PRF waveform. Without loss of generality, we apply two commonly used types of signal processing methods to verify that the designed waveform has good adaptability to different signal processing methods. And the two types of methods are based on the DFT method and compressed sensing (CS) theory; namely DFT-based method and CS-based method in the following, respectively.

#### 3.4.1. DFT-Based Signal Processing

**Figure 8:**
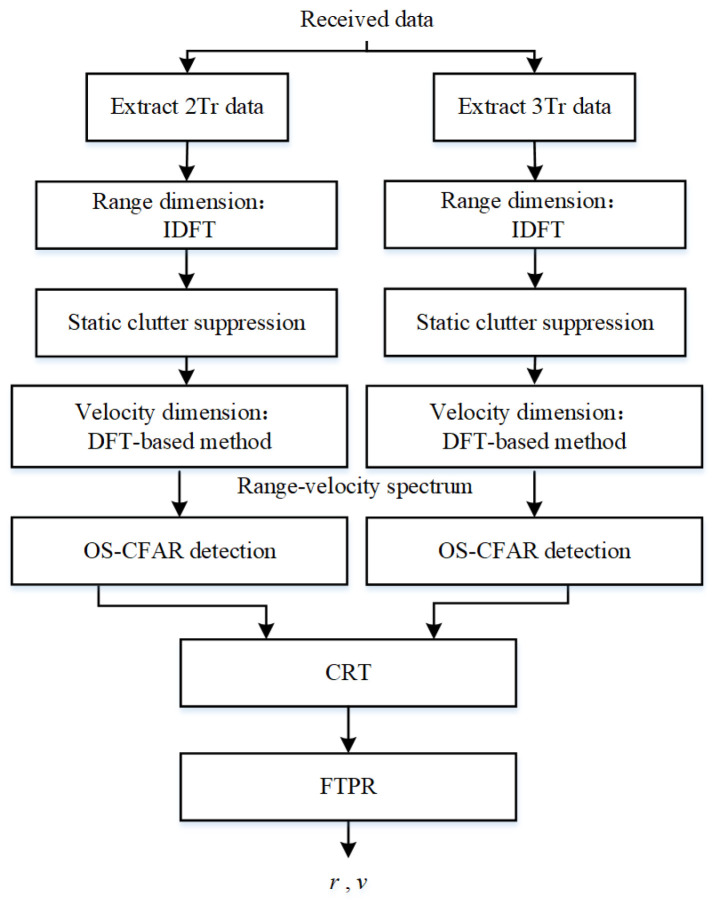
DFT-based signal processing.

Without loss of generality, the designed waveform can work with common signal processing methods such as the DFT-based method and the sparse recovery method. As we know, the designed dual-PRF waveform includes two uniform waveforms with different PRFs. So we can extract the two uniform waveforms from the overall sensing waveform and apply the DFT-based method to realize the parameter estimation. The DFT-based signal processing framework is illustrated in Figure 8.

First, it needs to extract 2Tr and 3Tr pulses data ($Y_{2Tr}$, $Y_{3Tr}$) according the sensing time from the nonuniform sensing receiving data. Taking the 2Tr pulses data for example, we apply DFT along each column of the data to obtain the range spectrum ${\overset{ˇ}{Y}}_{2Tr}=\left[{\overset{ˇ}{y}}_{2Tr}\left(0\right),\ldots ,{\overset{ˇ}{y}}_{2Tr}\left(N_{c}-1\right)\right]$, where ${\overset{ˇ}{y}}_{2Tr}\left(n\right)$ is the nth range cell.

Then, we apply the static clutter suppression along the slow-time dimension to each range cell, as given by

(24) $${\overset{ˇ}{y}}_{2Tr}\left(n\right)={\overset{ˇ}{y}}_{2Tr}\left(n\right)-\frac{1}{N_{s}}\sum {\overset{ˇ}{y}}_{2Tr}\left(n\right),$$

where $\sum {\overset{ˇ}{y}}_{2Tr}\left(n\right)$ denotes the element summation of ${\overset{ˇ}{y}}_{2Tr}\left(n\right)$. After that, we estimate velocity v along the slow-time dimension by the DFT method in order to obtain the range-velocity sepctrum ${\overset{ˇ}{Y}}_{2Tr}$ under uplink-flexible mode. Specially, we use the non-uniform DFT (NUDFT) method [34] to estimate the velocity of the dual-PRF sensing pulse under uplink-fixed mode because its extracting pulse is not strictly uniform timing. And the function of NUDFT is

(25) $$X\left(\omega_{n}\right)=\sum_{k=0}^{K-1}x\left(t_{k}\right)e^{-j\frac{2\pi }{K}nt_{k}},$$

where $x(t_{k})$ is the *k*th sample data, and $X(\omega_{n})$ is the *n*th NUDFT value. Above all, the IDFT method, the DFT method, and the NUDFT method are all based on DFT theory, so we call them all the DFT-based method.

Next, applying the OS-CFAR detector [35] to ${\overset{ˇ}{Y}}_{2Tr}$, it can detect the reflected objects. And the steps of the OS-CFAR detector are as follows: (1) select some protection cells around the ${\overset{ˇ}{Y}}_{2Tr}\left(n,k\right)$ cell to be detected; (2) select reference cells next to the protection cells and sort them by power, and select the middle one as the power of clutter and noise $P_{CN}$. (3) compute the threshold $T_{cfar}=z*P_{CN}$, where *z* is calculated without objects reflected, and (4) judge the existence of objects. If the power of the ${\overset{ˇ}{Y}}_{2Tr}\left(n,k\right)$ cell is greater than $T_{cfar}$, that means it is an object cell, otherwise, it is a noise cell. Applying the same steps above to all 3Tr pulses data, we can obtain the range-velocity spectrum of it.

Finally, we solve the velocity ambiguity using the CRT method [36] and computing the velocity using the frequency-time phase regression (FTPR) [37] method. According to the theory analysis and the ambiguity function of the designed waveform, we know that the velocity estimation of the 2Tr and 3Tr sensing pulses exhibit ambiguity values. We write their velocity values as ${\left\{v_{2\mathrm{Tr}}^{p}\right\}}_{p=0}^{P-1}$ and ${\left\{v_{3\mathrm{Tr}}^{q}\right\}}_{q=0}^{Q-1}$ including P and Q ambiguity values, respectively. Then we calculate the absolute differences of the two groups of ambiguity values

(26) $$\Delta {\left\{v_{na}\right\}}_{na=0}^{PQ-1}=\left|{\left\{v_{2\mathrm{Tr}}^{p}\right\}}_{p=0}^{P-1}-{\left\{v_{3\mathrm{Tr}}^{q}\right\}}_{q=0}^{Q-1}\right|.$$

Obviously, PQ is the total number of absolute differences and the unambiguity velocity is the group which has the minimum absolute differences. Considering the ${\overset{ˇ}{Y}}_{n,k}$ cell is an object cell, we then extract the spectrum of the nth range cell $\overset{ˇ}{y}\left(n\right)$ and set the spectrum zeros except for the $\left\{k\mathrm{th}-1,k\mathrm{th},k\mathrm{th}+1\right\}$ cells. Apply IDFT to the spectrum and rewrite it as ${\overset{ˇ}{y}}^{{}^{\prime }}\left(n\right)$, then calculate the Doppler of the object by

(27) $$f_{dt}=\frac{angle\left({\overset{ˇ}{y}}^{{}^{\prime }}\left(n,ka\right)\right)-angle\left({\overset{ˇ}{y}}^{{}^{\prime }}\left(n,ka\right)\right)}{2\pi (ka-kb)},$$

where angle() is a function of extracting phase, ka is the position of the maximum phase, and kb is the position of the minimum phase nearest the maximum one. Then, we can conveniently calculate the velocity of the object according to (3). Similarly, applying the FTPR method to the range dimension, we can also calculate the range of the object.

#### 3.4.2. CS-Based Signal Processing

**Figure 9:**
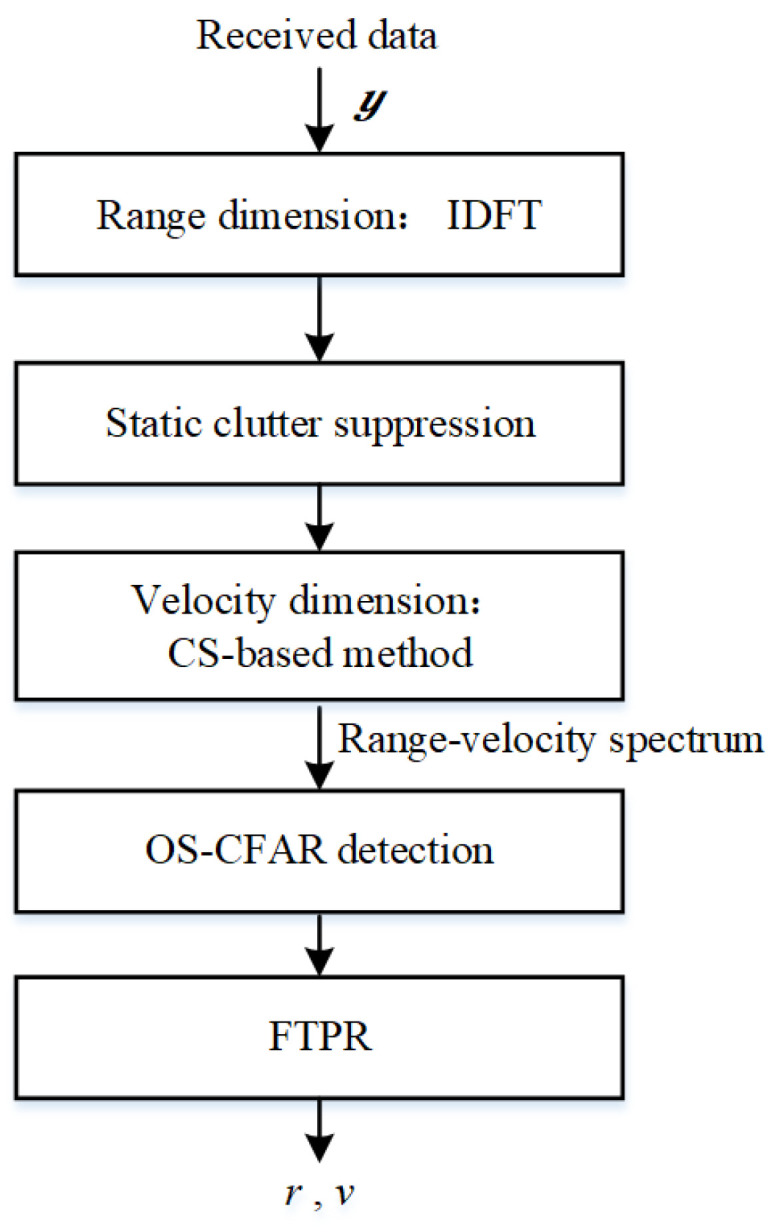
CS-based signal processing.

According to CS theory, if a signal is sparse or compressible in a certain transform domain, it may be reconstructed using nonlinear methods by solving an optimization problem with high probability. It is clear that the designed waveform is sparsely sampled in the Doppler dimension. We also apply the CS-based sensing signal processing to the designed waveform, and its framework is illustrated in Figure 9.

According to the Figure, we can see that the CS-based methods have the same pocessing steps on range spectrum, static clutter suppression, OS-CFAR detection, and FTPR. However, different from the DFT-based method, the CS-based methods do not need to solve velocity ambiguity. And here, we introduce two CS-based methods, namely the iterative adaptive approach (IAA) [38] and the orthogonal matching pursuit (OMP) [39] to reconstruct y.

The IAA algorithm solves the following optimization problem

(28) $$\min\;||y-\gamma_{k}a\left(f_{k}\right){\left|\right|}_{Q_{k}^{-1}}^{2}$$

where ${\left||x|\right|}_{Q_{k}^{-1}}^{2}=x^{H}Q_{k}^{-1}x$, $Q_{k}$ denotes the covariance matrix of the clutter and noise, and $|\gamma_{k}{|}^{2}$ is the power of the Doppler spectrum with frequency $f_{k}$. And the IAA method operates in an iterative way. The OMP method also operates in an iterative way, and the solution estimated at each iteration requires the solution of a least-squares problem, given by

(29) $$\min{∥A_{\Omega (p+1)}y_{\Omega (p+1)}-\hat{y}∥}_{2}^{2},$$

where $\Omega \left(p+1\right)=\{b\left(p_{i}+1\right)|p_{i}=1,2,\ldots ,p\}$ denotes the set consisting of b(p+1) for each iteration. The details of the IAA and the method can be seen in reference [12].

Then, we discuss the computation complexity of the three signal processing method. According to the designed timing, the designed sensing waveform is time-division and the range dimension is uniform, and we use the DFT method to estimate range. Hence, we focus on discussing the computational complexity of the DFT-based, IAA, and OMP methods. In this paper, we use fast Fourier transform (FFT) to realize the DFT-based method with a computational complexity of O(Llog(K)). The complexity of IAA and OMP are $O(L^{3}K^{3}))$ and $O(L^{2}K))$, respectively, and we set the number of iterations as both not more than 10 times in this paper.

## 4. Experimental Results

In this section, we illustrate the estimated performance of designed waveforms using simulated data and real data in traffic scenarios. We compare the performance of three waveforms (uniform sensing, dual-PRF under uplink-flexible, and dual-PRF under uplink-fixed mode) with the DFT-based method, the IAA method, and the OMP method.

### 4.1. Simulation Results

**Figure 10:**
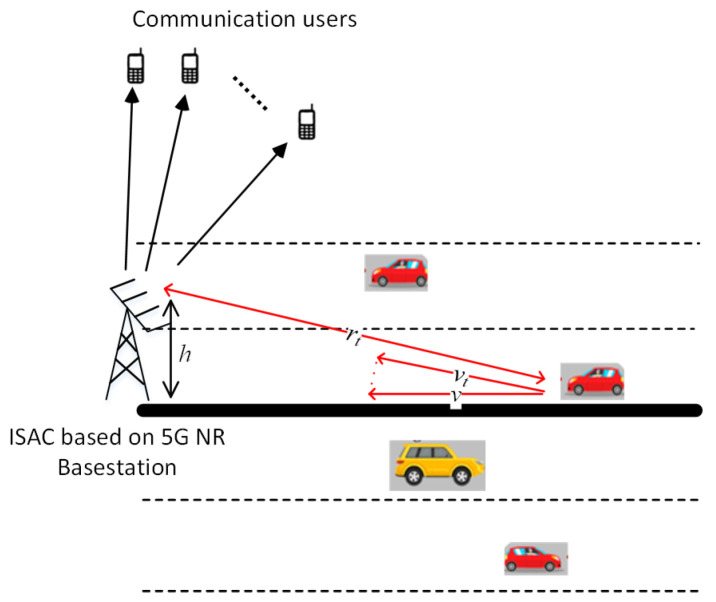
Traffic scenarios of the simulation experiments.

In this section, some simulation experiments are shown under a 5G communication base station. The traffic scenarios of the simulation experiments are shown in Figure 10, where *h* denotes the height of the base station, *v* denotes the forward velocity of the vehicle, $v_{t}$ denotes the radial velocity of the vehicle, and $r_{t}$ denotes the radial range of the vehicle. In the simulation experiment, it assumes that carrier frequency $f_{c}$ = 26 GHz, bandwidth B = 400 MHz, and subcarrier interval Δf = 120 kHz.

#### 4.1.1. One Vehicle

In the first simulation experiment, we illustrate the sensing effectiveness of the designed waveform and uniform waveform by the DFT-based method. Consider one car coming towards the communication station with r=100 m and v=60 km/h.

**Figure 11:**
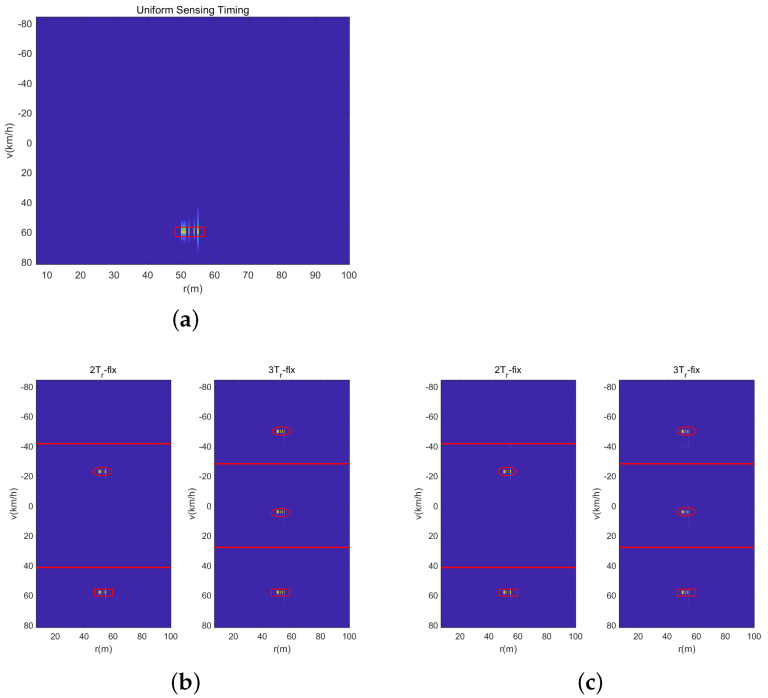
Estimated range-velocity spectrum. (**a**) Uniform Waveform. (**b**) Dual-PRF under uplink-flexible mode. (**c**) Dual-PRF under uplink-flexible mode.

Figure 11 shows the range-velocity map of three waveforms with the DFT-based method, where the red rectangle presents the real parameter of the real car, the red ellipse presents the ambiguous parameter, and the red line presents the unambiguity velocity region. Obviously, the uniform sensing waveform can detect the object unambiguously. In Figure 11b,c, we can see that the range-velocity map of the 2$T_{r}$ and 3$T_{r}$ sensing waveforms shows velocity ambiguously. Fortunately, the real velocity has the minimum bias between the range-velocity map of 2$T_{r}$ and 3$T_{r}$. Hence, it can solve the real velocity with the CRT method easily. Obviously, it can realize traffic detection and parameter estimation with the corresponding signal processing method under the designed sensing waveform.

Then, in the next simulation experiment, we illustrate the estimated performance of the designed sensing waveform with different signal processing methods. It assumes that the parameters of the detected car are the same as the last experiment, and 500 independent Monte Carlo experiments are performed under different signal-to-clutter-plus-noise ratios (SCNRs). The SCNR is defined as

(30) $$\begin{array}{c} \begin{array}{c} \mathrm{SCNR}=10lg\frac{P_{s}}{P_{c}+P_{n}}, \end{array} \end{array}$$

where $P_{s}$, $P_{c}$, $P_{n}$ denote the power of signal, clutter, and noise, respectively.

**Figure 12:**
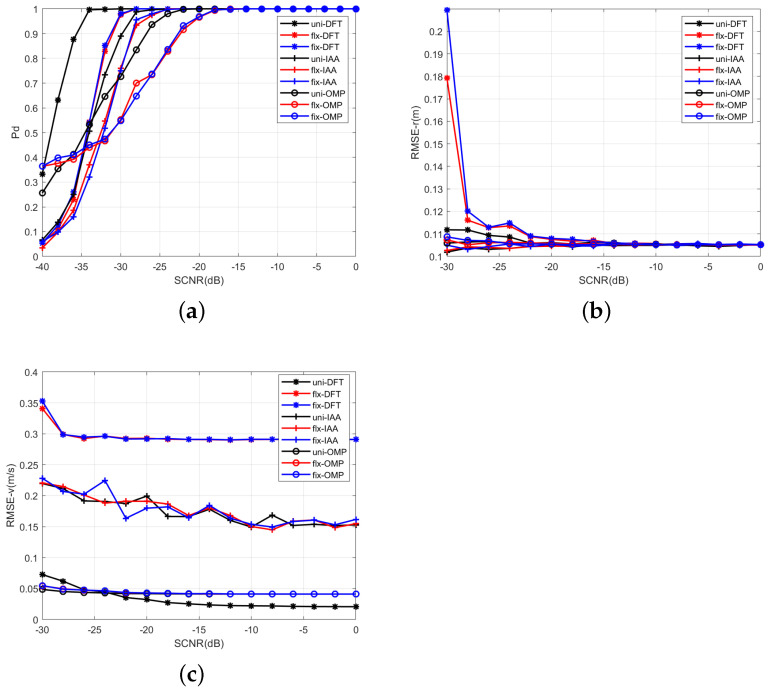
Performance Comparisons. (**a**) Detection performance under different SCNRs. (**b**) RMSEs of range. (**c**) RMSEs of velocity.

Figure 12a is the detection probability of the designed waveform with the DFT, IAA, and OMP methods. It can been seen that the probability of detection (Pd) increases with the increase in SCNR, and every signal processing method can achieve Pd > 95% while SCNR ≥−20 dB. The detection probability of the designed dual-PRF sensing waveform has some SCNR loss compared with that of uniform sensing. It can be seen that the detection probability of the designed dual-PRF sensing waveform has 5dB SCNR loss compared with that of uniform sensing with Pd = 0.9. And it is caused by less sensing symbols of the designed waveforms than that of the uniform one. Figure 12b,c illustrates the RMSEs of range and velocity, respectively. It can be seen from Figure 12b that the RMSE of the range tends to be consistent under every waveform because of the same range estimation method for each waveform. Unlike range estimation, the RMSE of velocity illustrates different results. It can be seen from Figure 12c that the estimation performance of the designed sensing waveform with the DFT method is poorer compared with the uniform one because of the use of less sensing symbols in the Doppler dimension. The velocity RMSE of the designed waveform is about 0.3 m/s (equal to 1.08 km/h) and it is one resolve cell Δv = 2.08 km/h. The RMSEs of velocity are almost similar under each sensing waveform with the IAA and the OMP method, respectively.

Actually, the IAA and the OMP methods improve estimation performance using nonlinear methods by solving an optimization problem. On one hand, they cost more in computing complexity. On the other hand, they need higher SCNR to realize accuracy reconstruction, and this can be verified in Figure 12a. It can be seen from the figure that there are obviously more SCNR loss by using the IAA and the OMP methods to the same waveform. It can be seen from the figure that there are obviously more SCNR losses by using the IAA and the OMP methods for the same waveform. For better or worse, the RMSEs of range and velocity are all in one solve cell, and these can satisfy the road traffic monitoring [40]. Hence, the designed sensing waveform can satisfy the traffic detection scenario with the common signal processing methods, such as the DFT, IAA, and OMP methods.

#### 4.1.2. Multiple Vehicles

**Figure 13:**
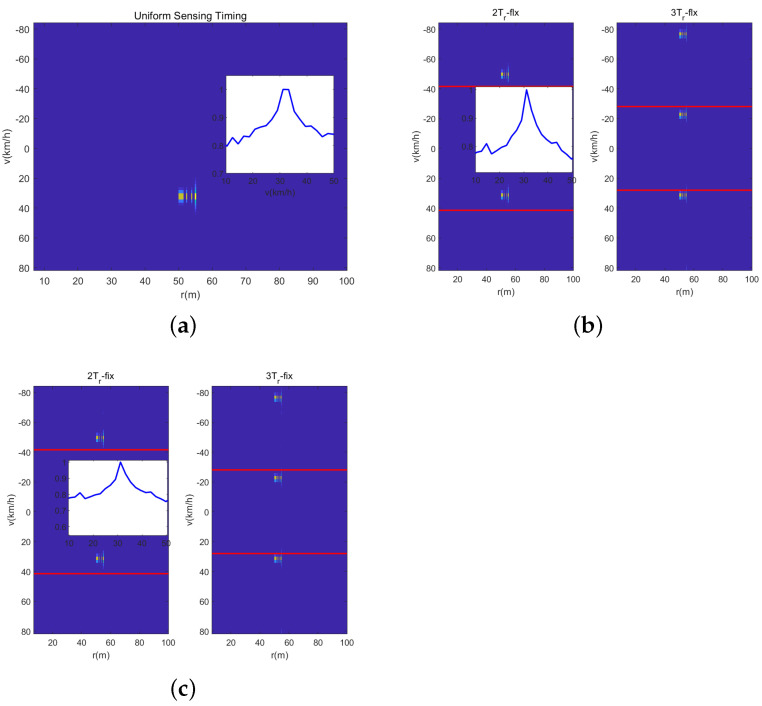
Vehicles solved by the DFT-based method. (**a**) Uniform Sensing. (**b**) Dual-PRF uplink-flexible mode. (**c**) Dual-PRF uplink-fixed mode.

**Figure 14:**
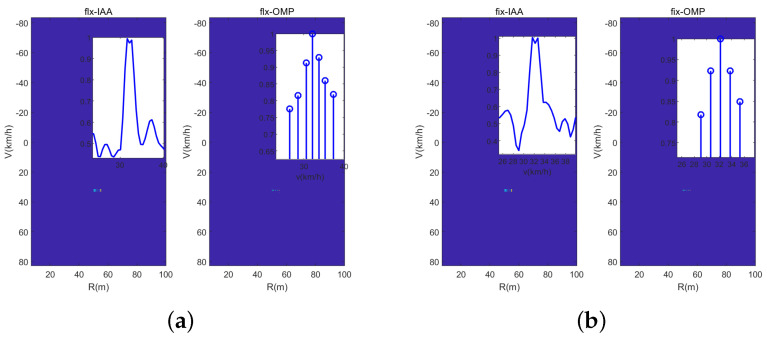
Vehicles solved by the IAA and OMP methods. (**a**) Dual-PRF uplink-flexible mode. (**b**) Dual-PRF uplink-fixed mode.

In the third experiment, we illustrate the solve performance of the designed sensing waveform with different signal processing methods. And we consider that there are two vehicles in one range cell with similar velocities of 31 km/h and 32 km/h. Figure 13 is the range-velocity map with the DFT-based method, wherein it can be seen from the velocity slice that neither sensing waveform can solve the two vehicles. The reason for this is that the velocity resolution Δv of the DFT-based method is 2.08 km/h. Figure 14 shows the range-velocity map using the IAA and OMP methods, and it can been seen that the IAA method can solve the two vehicles; otherwise, the OMP method cannot do it. In short, the IAA method has the best solve performance compared with the DFT-based method and the OMP method; otherwise, it has the highest computing complexity. Fortunately, in real common traffic scenarios, the case of two vehicles with similar range and velocity rarely occurs, and even if it occurs, it will not last for a long time. Hence, the designed waveform with the DFT-based method can satisfy the common traffic scenarios, and the experiments of real traffic scenarios in the next section will verify it.

### 4.2. Experiment Results in Real Traffic Scenarios

In this section, we illustrate some experiment results in real traffic scenarios. The sensing waveform is designed under the time division mode, and the sensing waveform looks like the radar pulse as shown in Figure 2. We thus use the radar module to emulate only the sensing waveform timing and verify its sensing performance. Furthermore, we use the IWR1642 radar module to imitate the sensing waveform of the TD-ISAC system. The radar module is not exactly the same with the 5G communication system, as we only use the radar to imitate the designed timing. One more issue to say is that there is CP in communication, but radar does not have it. Generally, in an ISAC system it removes the CP first while receiving the sampled signal, and then processes the signal without CP [41]. Hence, we use the radar module to imitate the sensing waveform without considering the CP.

**Figure 15:**
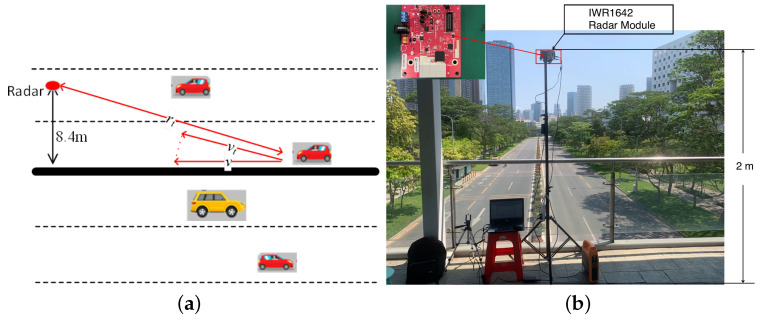
Real test of traffic scenarios. (**a**) Test scenarios of the radar installation. (**b**) Real traffic scenarios.

**Figure 16:**
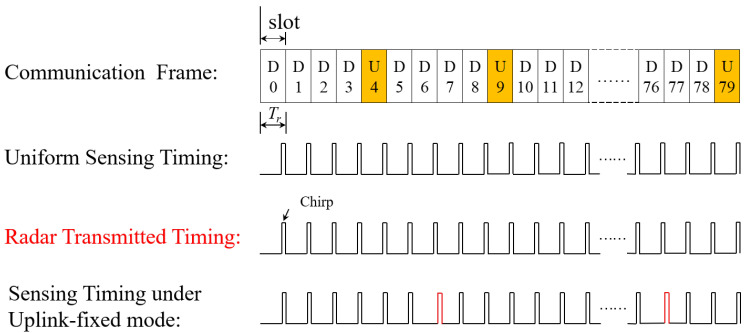
Sensing symbol timing and radar transmitted timing.

Figure 15 illustrate the real test of traffic scenarios. The IWR1642 radar is produced by Texas Instruments and operates with the carrier frequency of $f_{c}$ = 77 GHz. The radar installation height is 8.4m. The specific radar parameters are set as follows: sampling frequency $f_{s}$ = 5000 kHz, pulse wide of a chirp $T_{c}$ = 40 μs, number of chirps in a frame is 128, bandwidth of the sampled radar signal *B* = 420 MHz, and a frequency slope of 14 MHz/μs. From the parameters, there are 128 uniform chirps during a frame. We extract consecutive 80 chirps to emulate the uniform communication slot timing as shown in Figure 16, and extract the dual-PRF sensing waveform according to the timing of the dual-PRF sensing waveform under uplink-flexible mode as shown in Figure 3. As we know, there is timing modification under uplink-fixed mode as shown in Figure 5, and the modification is realized by shifting only to a OFDM symbol. However, the radar works in chirp mode, and there is no signal transmitted between two chirps. It is illustrated in Figure 16 that the modification timing (red pulse) under uplink-fixed mode cannot be emulated by the radar module. Hence, it cannot emulate the designed waveform under the uplink-fixed mode when limited to its parameters. We only compare the sensing performance of the uniform waveform with the dual-PRF sensing waveform under uplink-flexible mode in real traffic scenarios.

**Figure 17:**
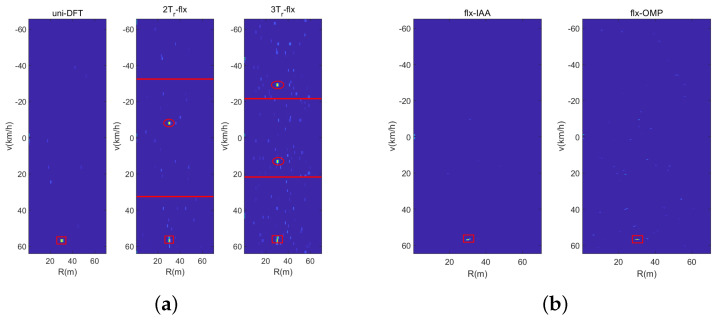
One vehicle in real traffic scenario. (**a**) DFT-based method. (**b**) IAA and OMP method.

Figure 17 illustrates the detected results for one vehicle, where the rectangle denotes that the detection cell is a real vehicle, and the ellipse denotes the ambiguity targets. It can be seen that the uniform sensing waveform with the DFT method and the dual-PRF sensing waveform under uplink-flexible mode with the IAA and OMP methods can all solve for the vehicle unambiguously. Although the $2T_{r}$ and $3T_{r}$ sensing pulses have ambiguous velocity with the DFT-based method, it can also solve for the real vehicle by the CRT method obviously.

**Figure 18:**
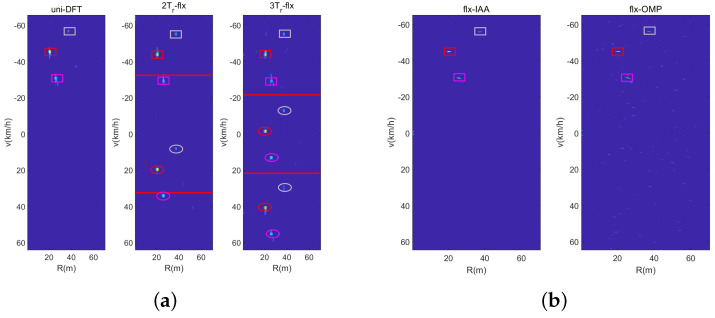
Multiple vehicles in real traffic scenario. (**a**) DFT-based method. (**b**) IAA and OMP method.

Figure 18 illustrates the detected results of multiple vehicles, where the different colors represent three vehicles, respectively. The shapes of rectangle and ellipse denote the same meaning as that in Figure 17. It has the same results as those of the last experiment in each range cell. Hence, the designed sensing waveform can realize the sensing of multiple vehicles with the three signal processing under real common traffic scenarios.

## 5. Conclusions

The millimeter wave band in 5G greatly reduces the time for each communication symbol. Hence, utilizing a small number of symbols in one frame for sensing can greatly alleviate the problem of low efficiency. In this paper, we design a TD-ISAC sensing waveform based on a 5G communication base station with its millimeter wave band. We design two dual-PRF sensing waveforms subject to uplink-flexible and uplink-fixed mode. More precisely, we allocate only one OFDM symbol for sensing in one time slot at most to deduce the impact on communication efficiency. And the designed waveforms occupy only 53 OFDM symbols, whereby it has 1120 OFDM symbols total in one communication frame. In fact, it usually has some reserved symbols in a communication frame which can be utilized for sensing. The sensing performance of the designed waveforms with the DFT-based method is a little poorer compared with that of the uniform sensing waveform. But we know that it cannot realize uniform sensing in 5G communication. The designed sensing waveform can also realize vehicle detection and estimation by applying the DFT-based method in real traffic scenarios. In addition, it can improve estimation performance by applying a sparse recovery method, e.g., the IAA and OMP method, at the expense of computational complexity. The designed dual-PRF waveform can work well under a general communication status. Our future research work will focus on designing sensing waveforms under burst communication services in order to be adaptive to any communication status.

## Data Availability

Data are contained within the article.
